# Corrigendum: PARP3 is a promoter of chromosomal rearrangements and limits G4 DNA

**DOI:** 10.1038/ncomms15918

**Published:** 2017-06-13

**Authors:** Tovah A. Day, Jacob V. Layer, J. Patrick Cleary, Srijoy Guha, Kristen E. Stevenson, Trevor Tivey, Sunhee Kim, Anna C Schinzel, Francesca Izzo, John Doench, David E. Root, William C. Hahn, Brendan D. Price, David M. Weinstock

Nature Communications 8: Article number: 15110; DOI: 10.1038/ncomms15110 (2017); Published: 04 27 2017; Updated: 06 13 2017

In this Article, the antibody ‘BG4’ is consistently referred to incorrectly as ‘hf2’. These errors appear in the Results and Methods. Additionally, the Article incorrectly cites reference 52 in the sentences ‘A recent study described hf2, an engineered single-chain antibody specific for G4 DNA^52^’ and ‘pSANG10-3F-BG4 (Addgene 55756)^52^ was transformed into….’ The correct citation is given below.

Biffi, G., Tannahill, D., McCafferty, J. & Balasubramanian, S. Quantitative visualization of DNA G-quadruplex structures in human cells. *Nat. Chem.* **5**, 182–186 (2013).

