# Supplementary material for: PARP3 is a promoter of chromosomal rearrangements and limits G4 DNA
Source: Nat Commun. 2017 Apr 27;8:15110. doi: 10.1038/ncomms15110 (PMC5414184; doi:10.1038/ncomms15110)
Supplement: Supplementary Information — Supplementary Figures, Supplementary Tables, Supplementary Methods, and Supplementary References [file ncomms15110-s1.pdf]

Supplementary Figure 1

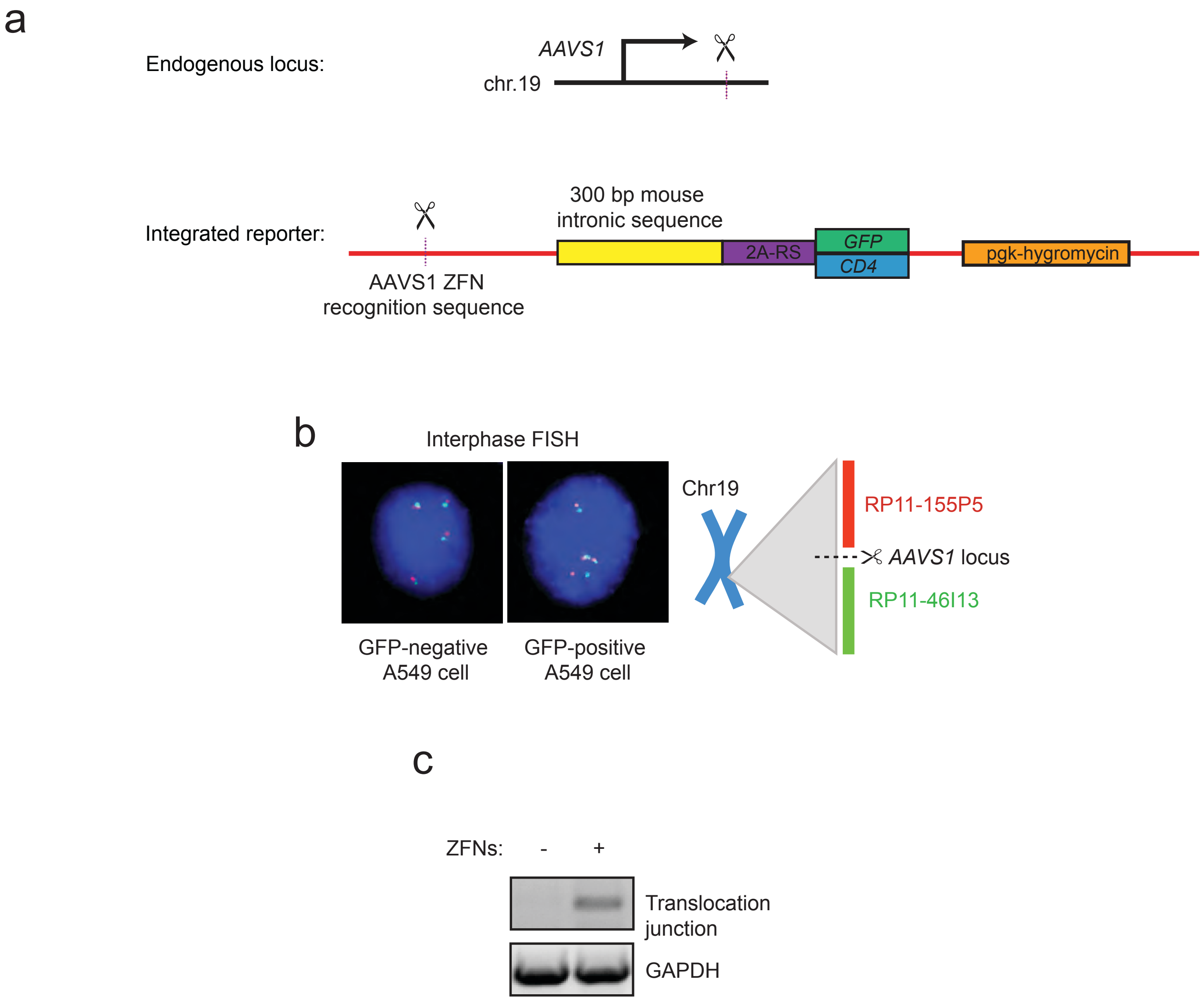

Supplementary Figure 1 | Validation of flow-based assay for chromosomal rearrangements. (a) Schematic of rearrangement reporter. 2A-RS, ribosomal stutter site. Pgk, phosphoglycerate kinase 1 promoter. (b) Interphase fluorescence in situ hybridization (FISH) of A549 GFP-negative and GFP-positive cells using a break-apart probe that spans the AAVS1 locus cleavage site. (c) PCR for rearrangement between the endogenous AAVS1 locus and the GFP-containing cassette after transduction with AAVS1 ZFNs.

Supplementary Figure 2

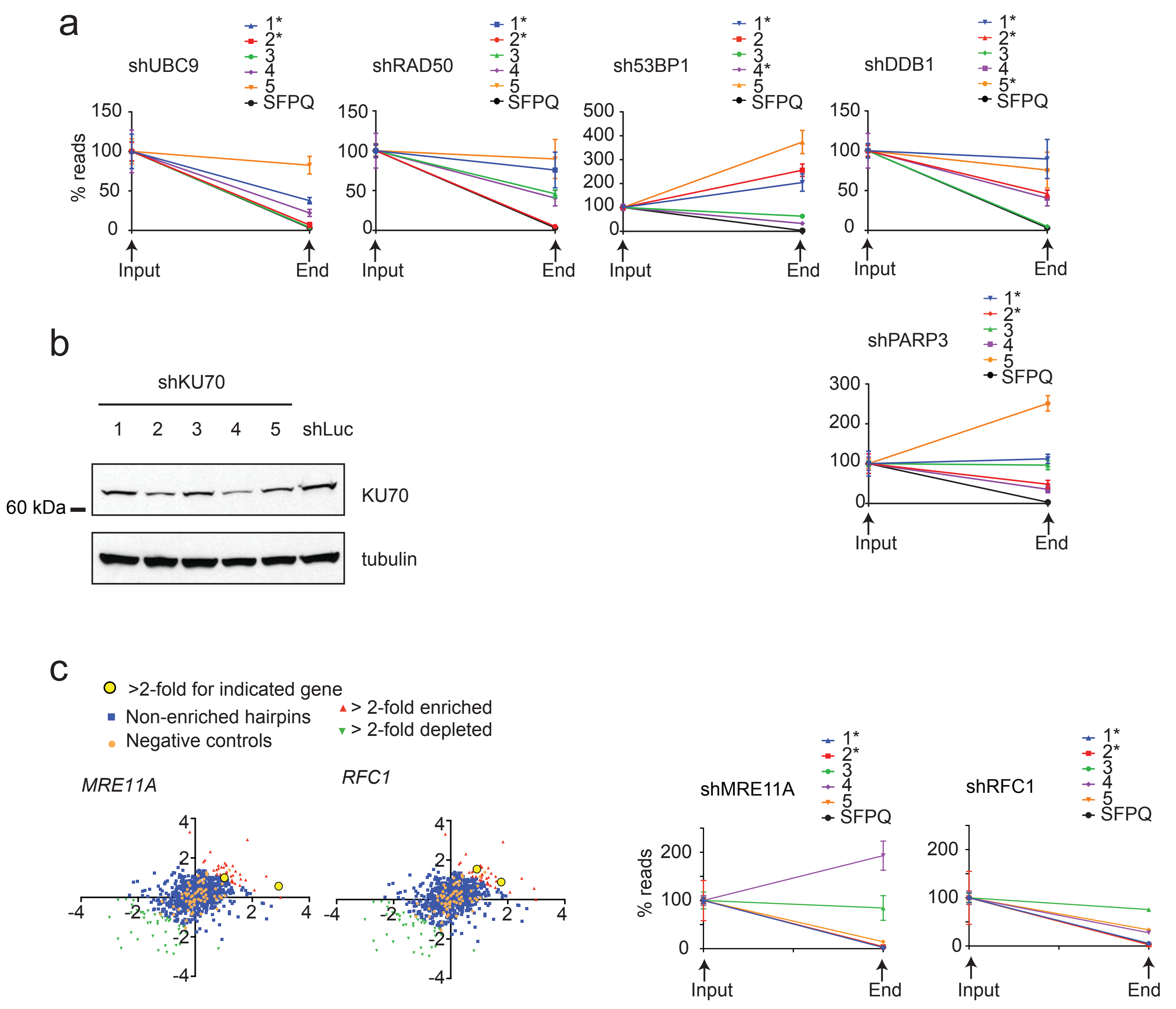

Supplementary Figure 2 | shRNA screen for factors that modulate rearrangement frequency identified 5 genes. (a) shRNA representation at initial transduction (input, Day 0) and prior to flow-sorting (end, Day 9) for shRNA targeting each of the 5 hits. To be considered for further analysis, at least one scoring hairpin was represented at ≥30% of input. SFPQ represents a control hairpin with significant toxicity in A549 cells. (b) Immunoblot of A549 cells transduced with lentivirus expressing the five different shRNA targeting KU70 included in the screen along with an shRNA targeting luciferase (luc) as a control. (c) Examples of genes with ≥2 hairpins with >2-fold effect on translocations (left) that were excluded due to the toxicity of the scoring hairpins. On the left, the average of $\log_2$ reads in transgene-positive population / average $\log_2$ reads in transgene-negative population for GFP replicates (x-axis) and CD4 replicates (y-axis) is shown. On the right, shRNA representation at initial transduction (input, Day 0) and prior to flow-sorting (end, Day 9) for shRNA is shown. shRNA that scored in the rearrangement screen are marked with an asterisk.

Supplementary Figure 3

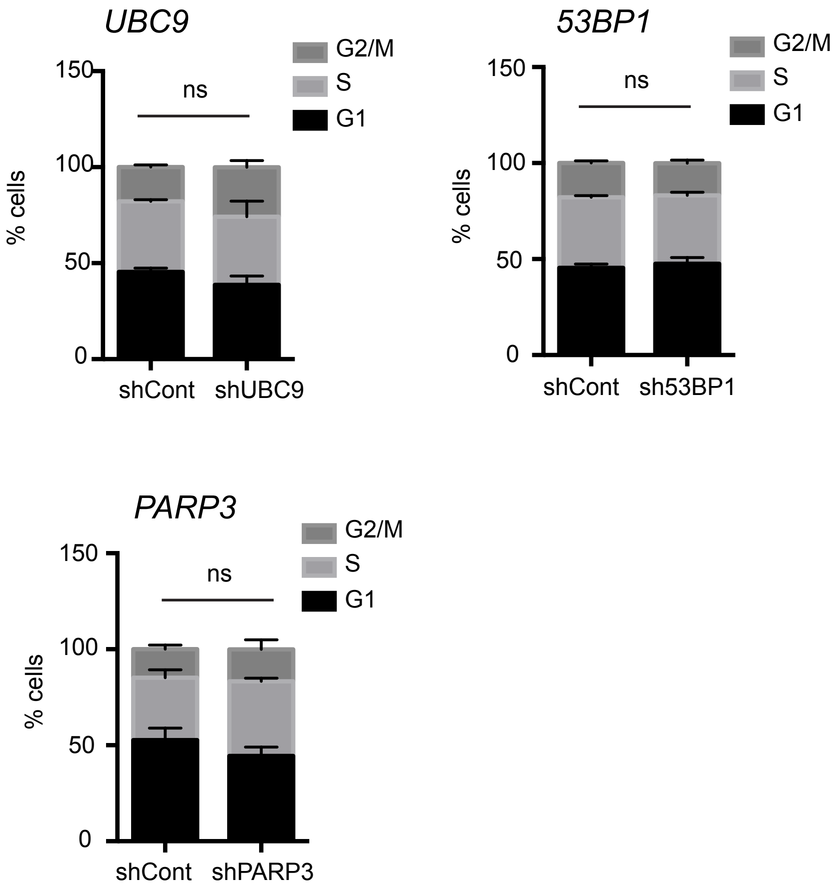

Supplementary Figure 3 | shRNA-mediated knockdown of hits from translocation factor screen does not significantly alter cell cycle distribution. Quantification of cell cycle distribution in HeLa cells stably transduced with shRNA directed at the indicated target or control shRNA and propagated in culture. Data represents mean ± SE.

Supplementary Figure 4

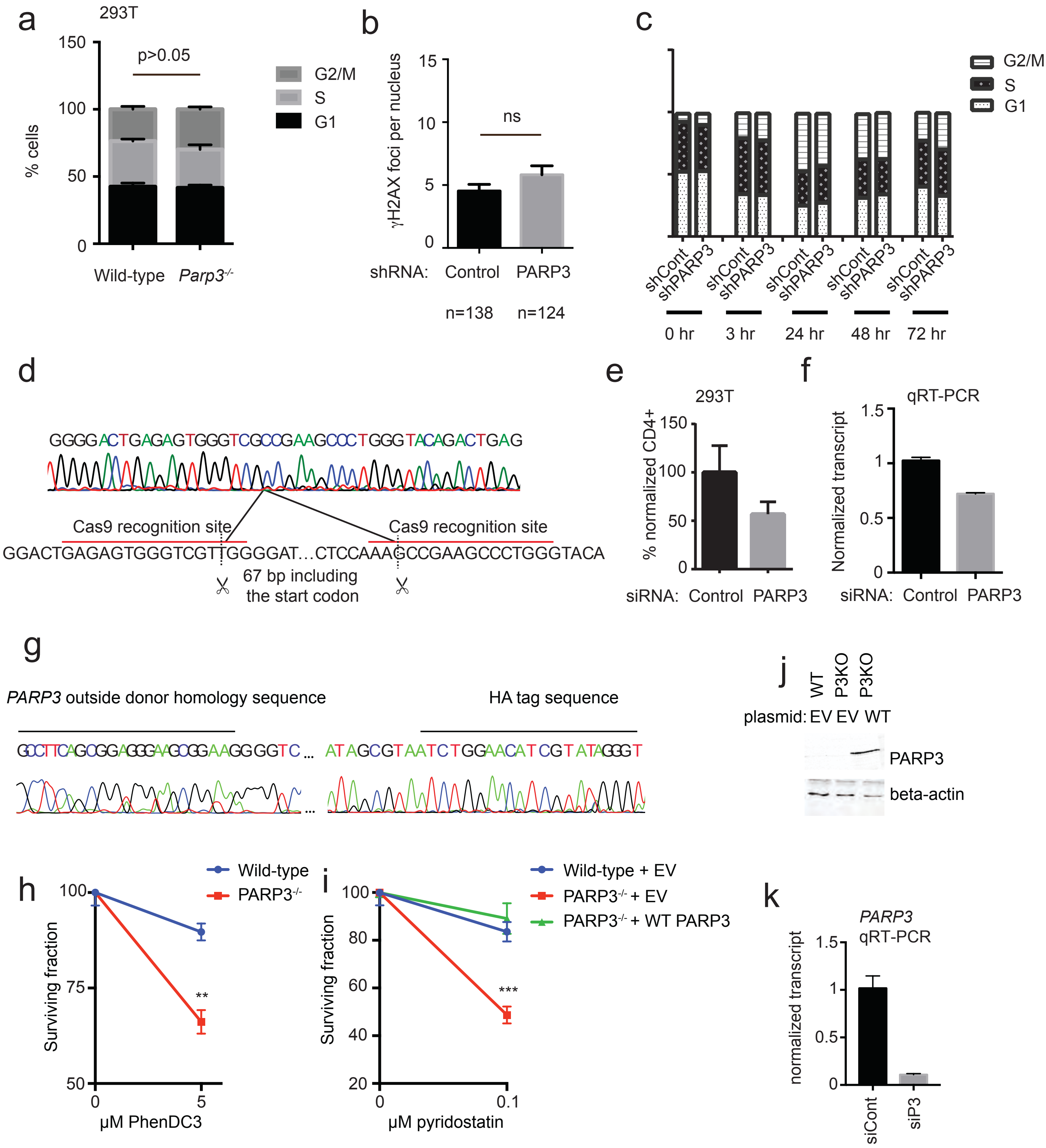

Supplementary Figure 4 | (a) Cell cycle distribution of HeLa cells stably transduced with shRNA directed at PARP3 or control shRNA. (b) Quantification of γH2AX foci per nucleus (n = number of nuclei) in HeLa cells expressing shRNA targeting PARP3 or non-targeting control. (c) Cell cycle distribution of HeLa cells expressing shRNA targeting PARP3 or Control (Cont) at the indicated time points following 1 Gy IR. (d) Sanger sequencing chromatogram of Cas9-mediated deletion in the first exon of human PARP3. Scissors, Cas9-mediated DSBs. (e,f) Normalized frequency of Cas9-mediated rearrangements between *CD71* and *CD4* (e) and quantification of PARP3 transcript (f) in 293T cells transfected with siRNA targeting PARP3. (g) Sanger sequencing chromatogram confirming targeted insertion of N-terminal HA-tag at the endogenous PARP3 locus in 293T cells. (h) Colony survival assay using wild-type and *PARP3*$^{-/-}$ A549 cells with the indicated dose of PhenDC3. (i,j) Colony survival assay (i) and immunoblot (j) with *PARP3*$^{-/-}$ A549 cells transfected with empty vector (EV) or wild-type PARP3 (WT PARP3)-expressing plasmid treated with the indicated dose of pyridostatin. (k) PARP3 transcript levels measured by qRT-PCR in A549 cells transfected with control siRNA (siCont) or targeting PARP3. P values calculated using unpaired Student's t test. *P < 0.05, **P < 0.01, ***P < 0.001. Data represents mean ± SE.

Supplementary Figure 5 | Heat map of differentially expressed genes (N=61) with adjusted *p* value<0.05 and $\log_2$ Fold-Change >1 of relative log-transformed values across vehicle-treated *PARP3*$^{-/-}$ vs. vehicle-treated wild-type A549 cells.

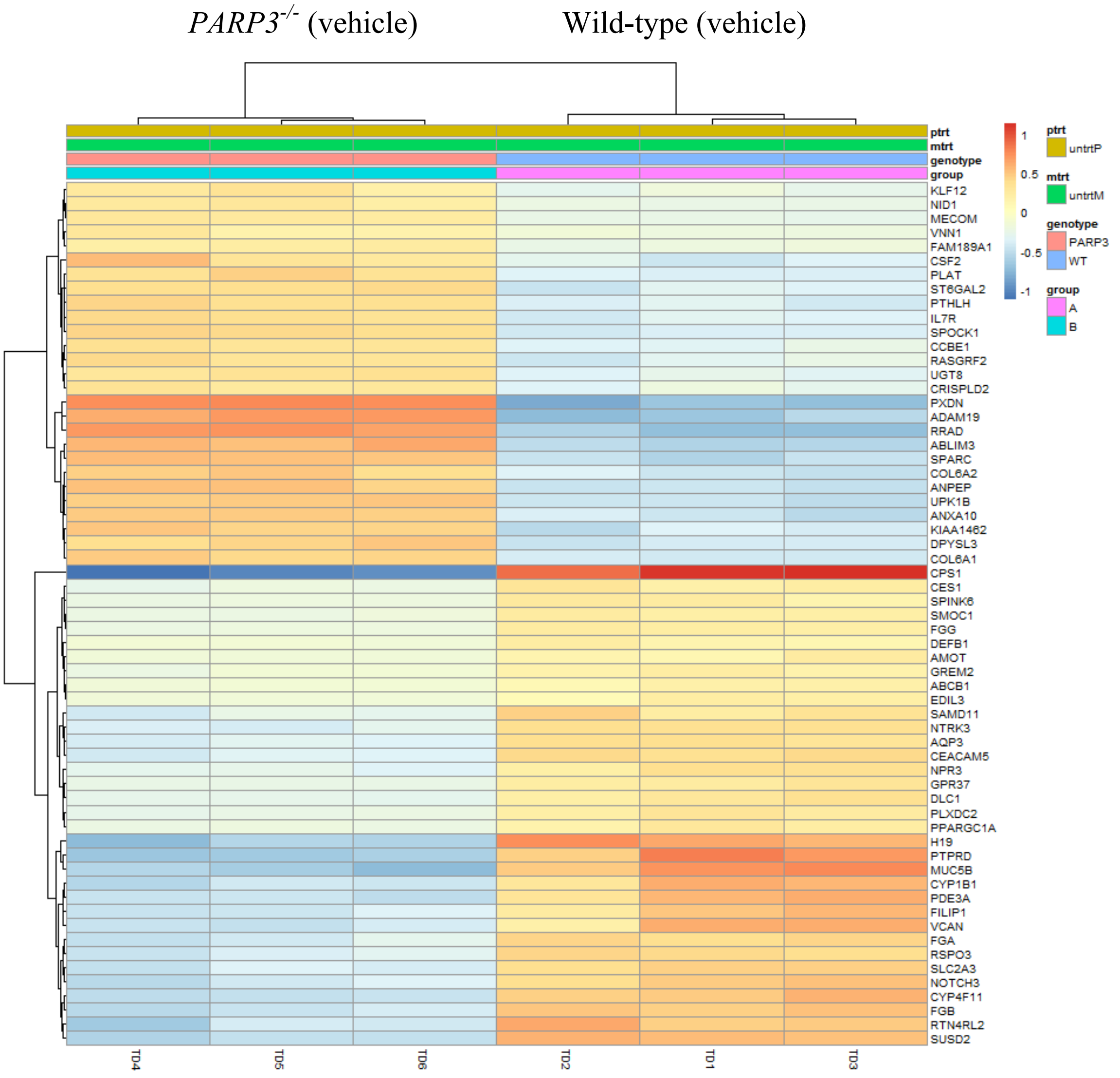

Supplementary Figure 6

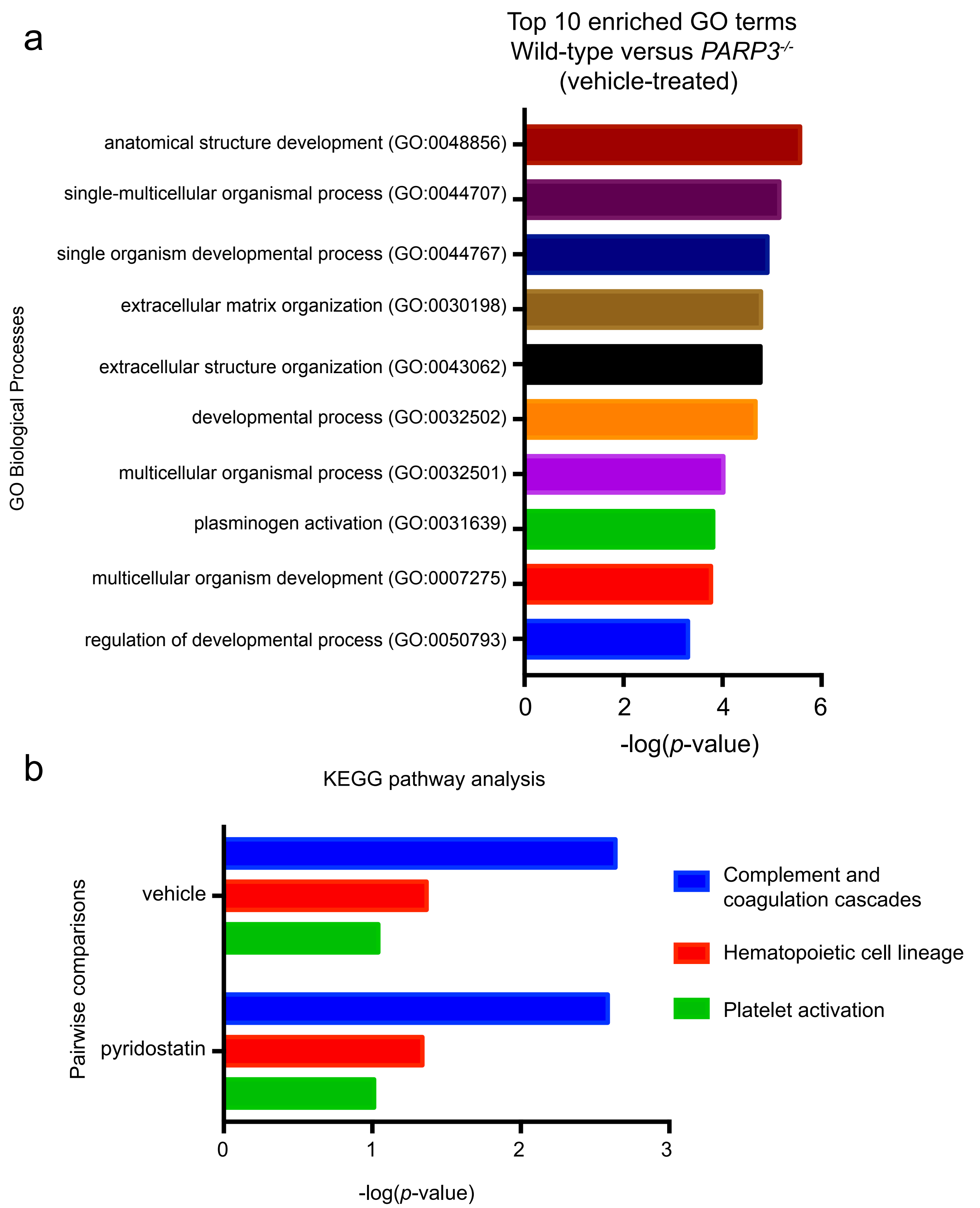

Supplementary Figure 6 | (a) Top 10 Gene Ontology (GO) enrichment terms in the 61 significantly differentially expressed genes between vehicle-treated wild-type and vehicle-treated *PARP3*$^{-/-}$ A549 cells. (b) KEGG pathway analysis showing pathways that were significantly enriched in the differentially expressed genes between wild-type and *PARP3*$^{-/-}$ cells with either vehicle treatment or pyridostatin treatment.

Supplementary Figure 7 | Heat map of differentially expressed genes (N=64) with adjusted *p* value <0.05 and $\log_2$ Fold-Change >1 of relative log-transformed values across pyridostatin-treated *PARP3*$^{-/-}$ vs. pyridostatin-treated wild-type A549 cells. Cells were treated with 1.0 μM pyridostatin for 24 hours prior to RNA collection.

PARP3$^{-/-}$ (pyridostatin) Wild-type (pyridostatin)

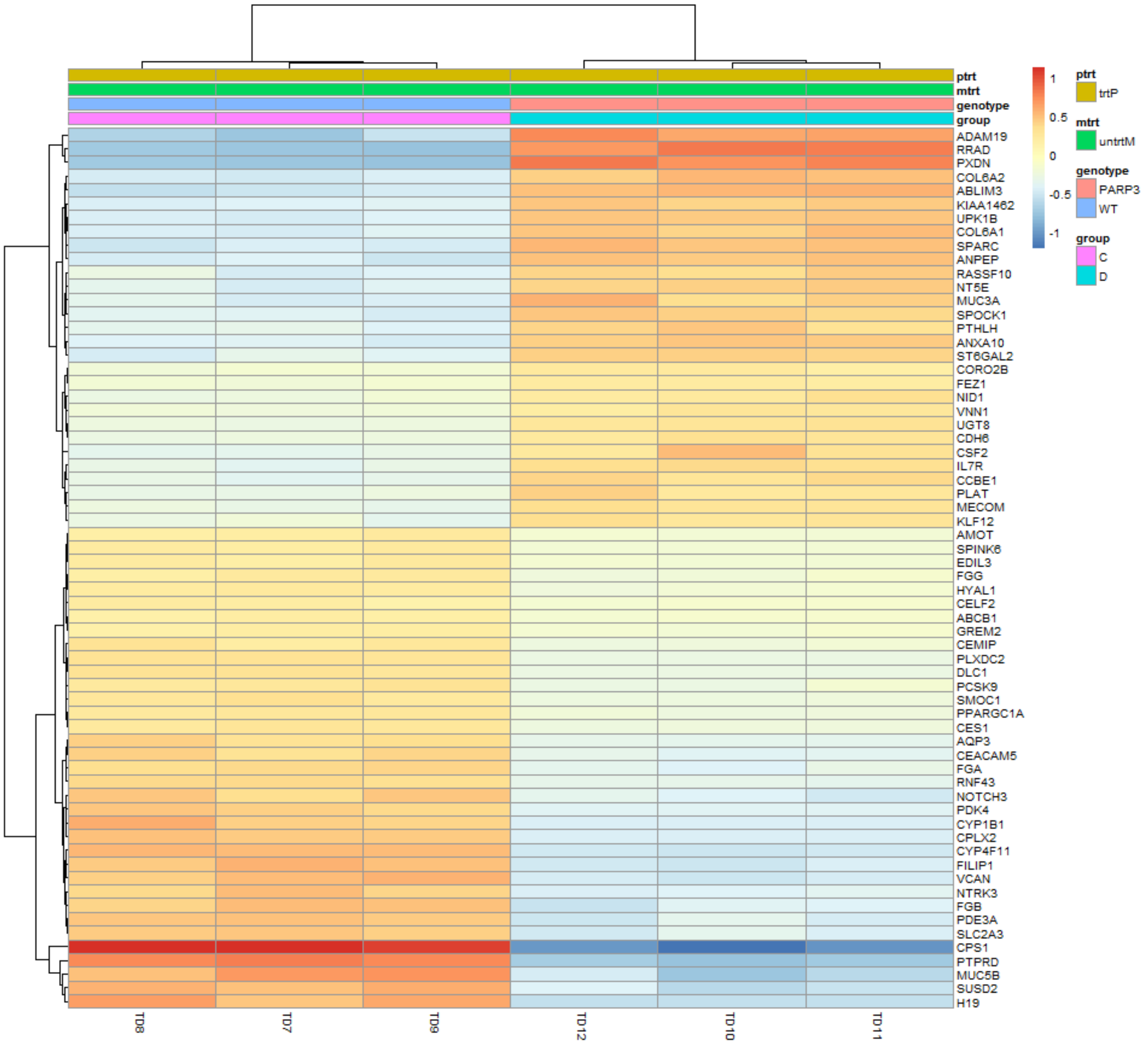

Supplementary Figure 8

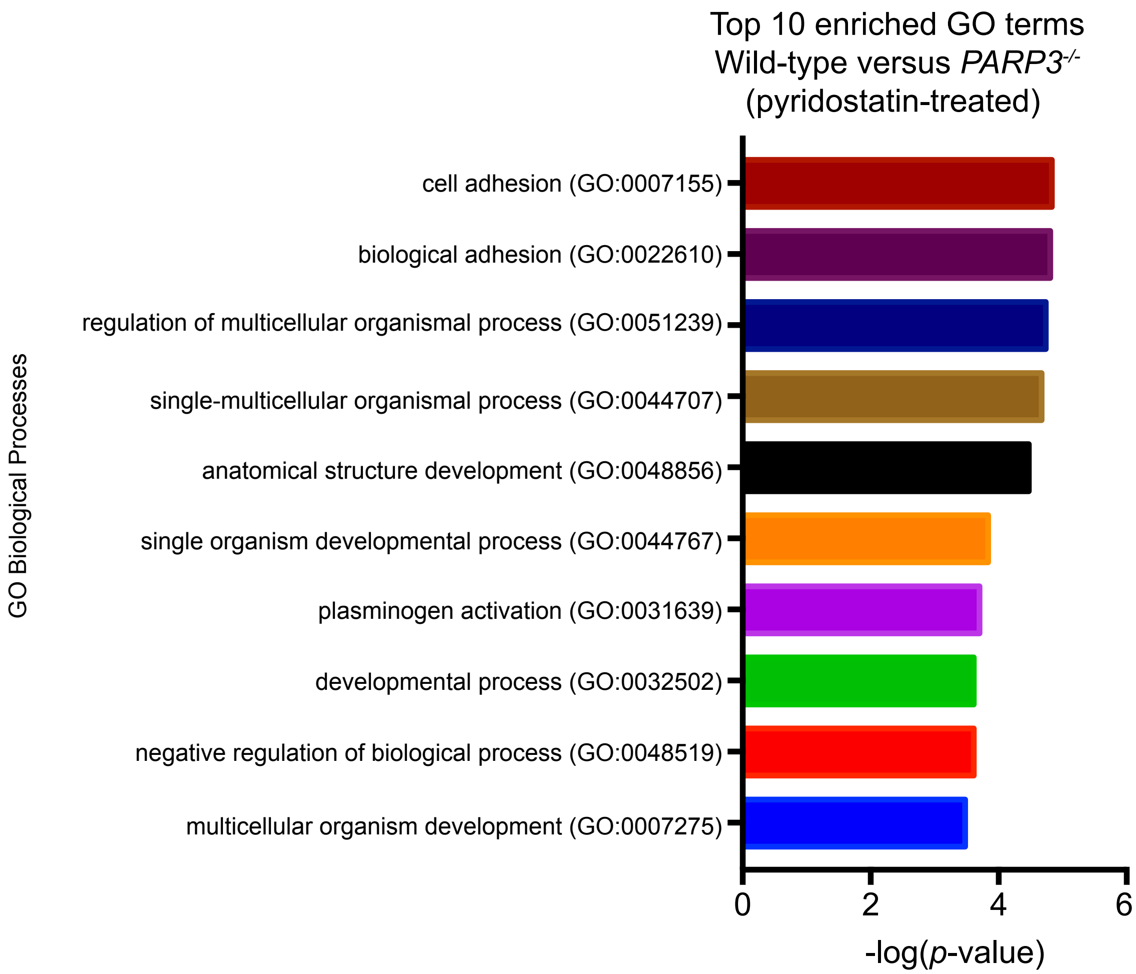

Supplementary Figure 8 | Top 10 Gene Ontology (GO) enrichment terms in the 64 genes significantly differentially expressed between pyridostatin-treated wild-type and pyridostatin-treated *PARP3*$^{-/-}$ A549 cells.

Supplementary Figure 9

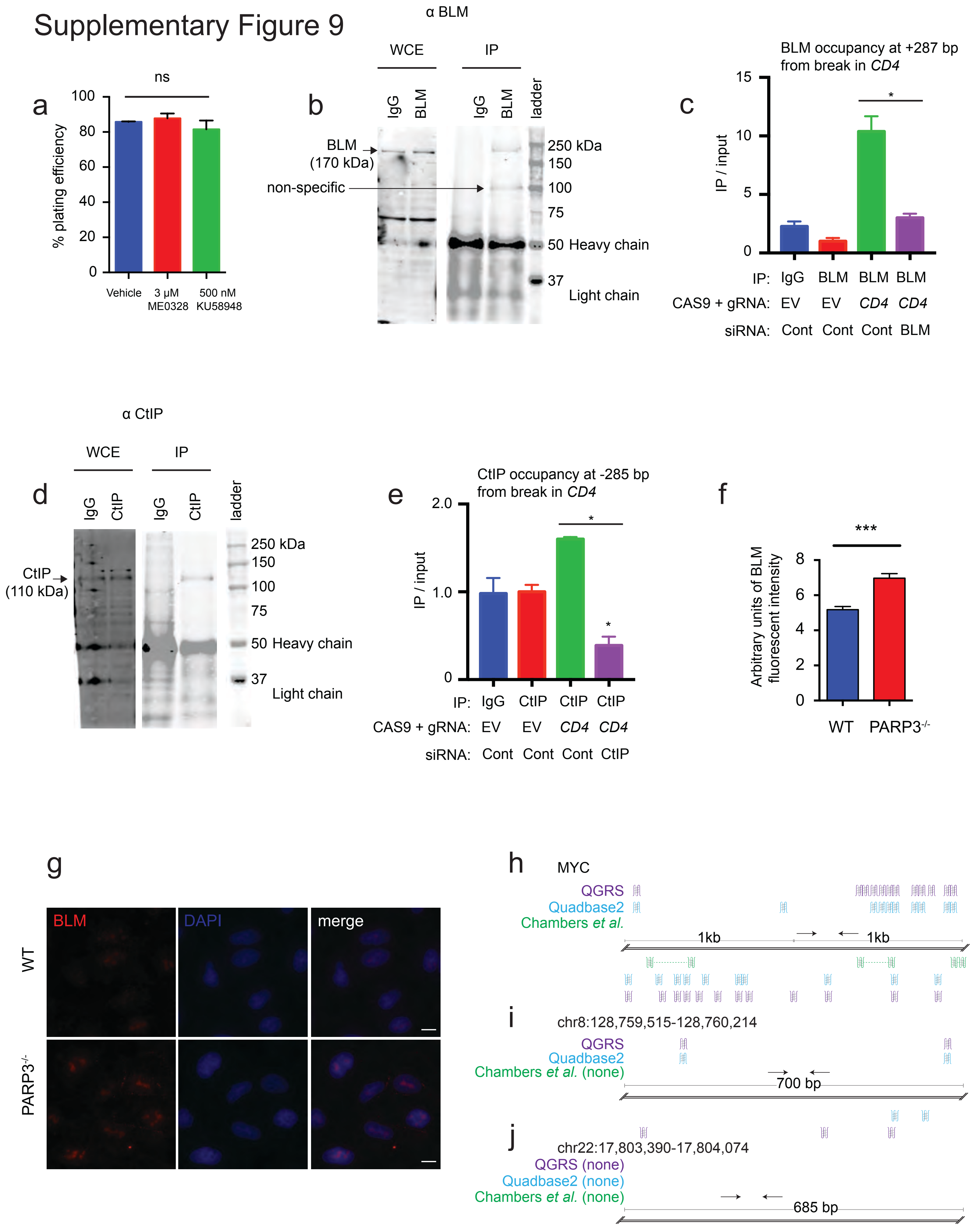

Supplementary Figure 9 | ME0328 toxicity, BLM accumulation on chromatin, and G4 DNA loci. (**a**) Quantification of percent survival of wild-type A549 cells treated with vehicle, the PARP3-specific inhibitor ME0328 (3 μM), or the pan-PARP1/PARP2/PARP3 inhibitor KU58948 (500 nM). (**b-e**) Immunoprecipitation (IP) and western blot with whole cell extracts (WCE) and IP for BLM (**b**) (predicted size 170 kDa) and CtIP (predicted size 110 kDa) (**d**) (IgG, immunoglobulin. kDa, kiloDaltons. Heavy chain, immunoglobulin heavy chain. Light chain, immunoglobulin light chain) and chromatin immunoprecipitation (ChIP) specificity controls for BLM (**c**) and CtIP (**e**) using siRNA and CRISPR/Cas9-mediated cutting at *CD4*. A single non-specific band is apparent in the BLM IP (**b**). Therefore, the demonstration that the BLM ChIP signal is reduced upon siBLM treatment is an important alternative specificity control (**c**). (**f,g**) Quantification of BLM staining using arbitrary units of fluorescence intensity (**f**) and representative images (**g**) of BLM immunofluorescence in wild-type (WT) and *PARP3*$^{-/-}$ A549 cells. Scale bar, 10 μM. (**h-j**) Schematic of sequences predicted by QGRS (purple) and Quadbase2 (blue) to form G4 DNA and sequences experimentally observed to form G4 DNA by Chambers *et al.*, 2015 (green) at the human *MYC* promoter (**h**), chr8:128,759,515-128,760,214 (**i**), and chr22: 17,803,390-17,804,074 (**j**). *P* values calculated using unpaired Student's t test. **P* < 0.05, ***P* < 0.01, ****P* < 0.001. Data represents mean ± SE.

Supplementary Figure 10

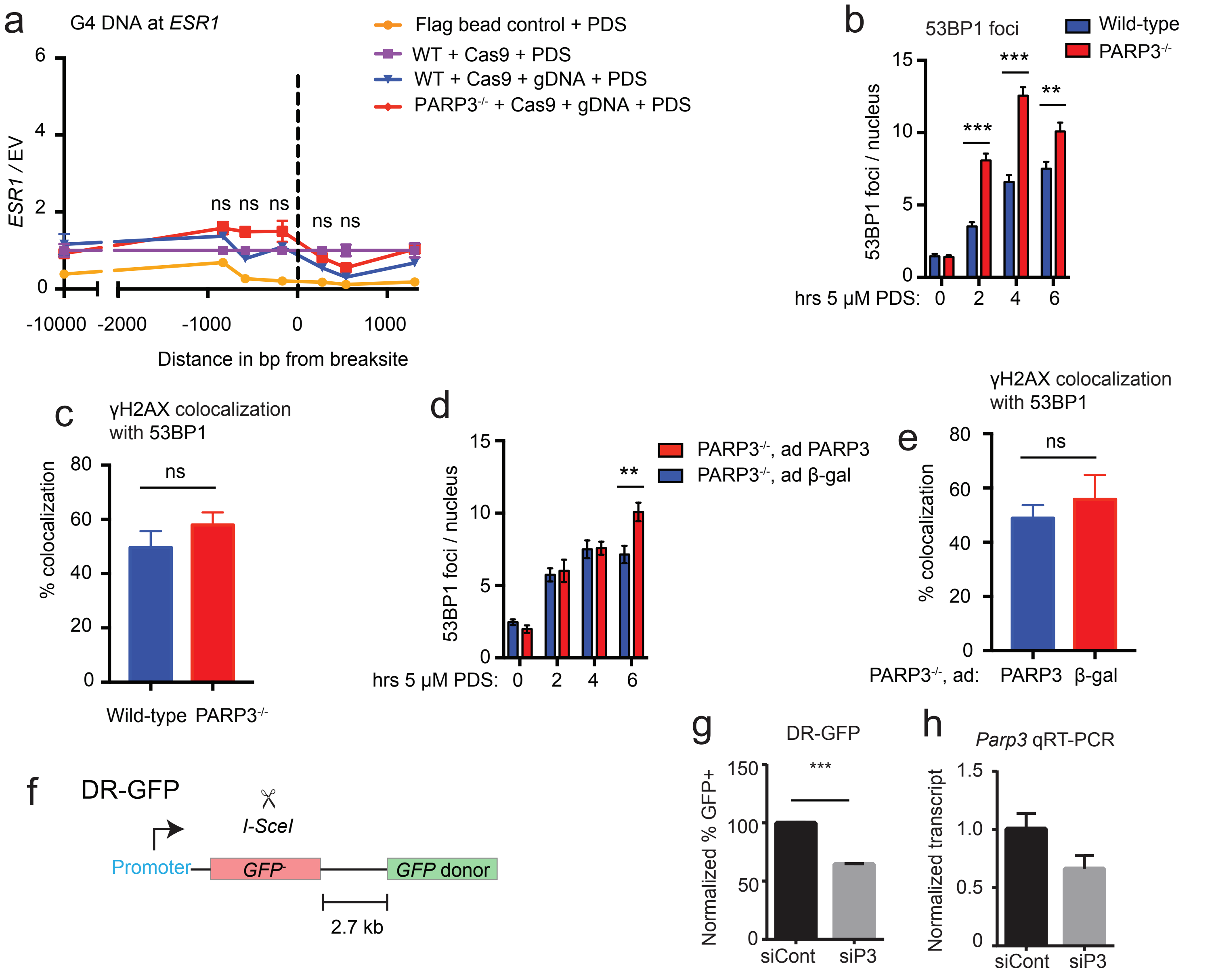

Supplementary Figure 10 | DSB repair in *PARP3*$^{-/-}$ cells. (a) Quantification of G4 DNA content in the indicated genotypes at the *ESR1* locus measured by immunoprecipitation (IP) with hf2 24 hours after pyridostatin (PDS) treatment and 18 hours after transfection with CRISPR/Cas9 with or without gRNA for *ESR1*. bp, base pairs. (b,c) Quantification of immunofluorescence (IF) for 53BP1 at the indicated time points following treatment with 5 µM PDS (b) and degree of colocalization between γH2AX and 53BP1 at 4 hours following 5 µM PDS (c) in wild-type and *PARP3*$^{-/-}$ A549 cells. (d,e) Quantification of IF for 53BP1 at the indicated time points following treatment with 5 µM PDS (d) and degree of colocalization between γH2AX and 53BP1 at 6 hours following 5 µM PDS (e) in *PARP3*$^{-/-}$ A549 cells infected with adenovirus expressing PARP3 (ad PARP3) or β-galactosidase (ad β-gal). (f) Schematic of the homologous recombination (HR) reporter DR-GFP, which involves cleavage within a nonfunctional GFP gene by the endonuclease I-SceI. Repair by HR using the downstream GFP donor template can result in a functional GFP gene and quantified by flow cytometry, (g) Normalized frequency of GFP-positive cells in U2OS cells containing DR-GFP 48 hours after transfection of I-SceI. (h) Quantification of PARP3 transcript 24 hours after transfection with siRNA targeting PARP3 (siP3) or Control (siCont). P values calculated using unpaired Student's t test. *$P < 0.05$, **$P < 0.01$, ***$P < 0.001$. Data represents mean ± SE.

Supplementary Figure 11

Scale bar is 10 microns

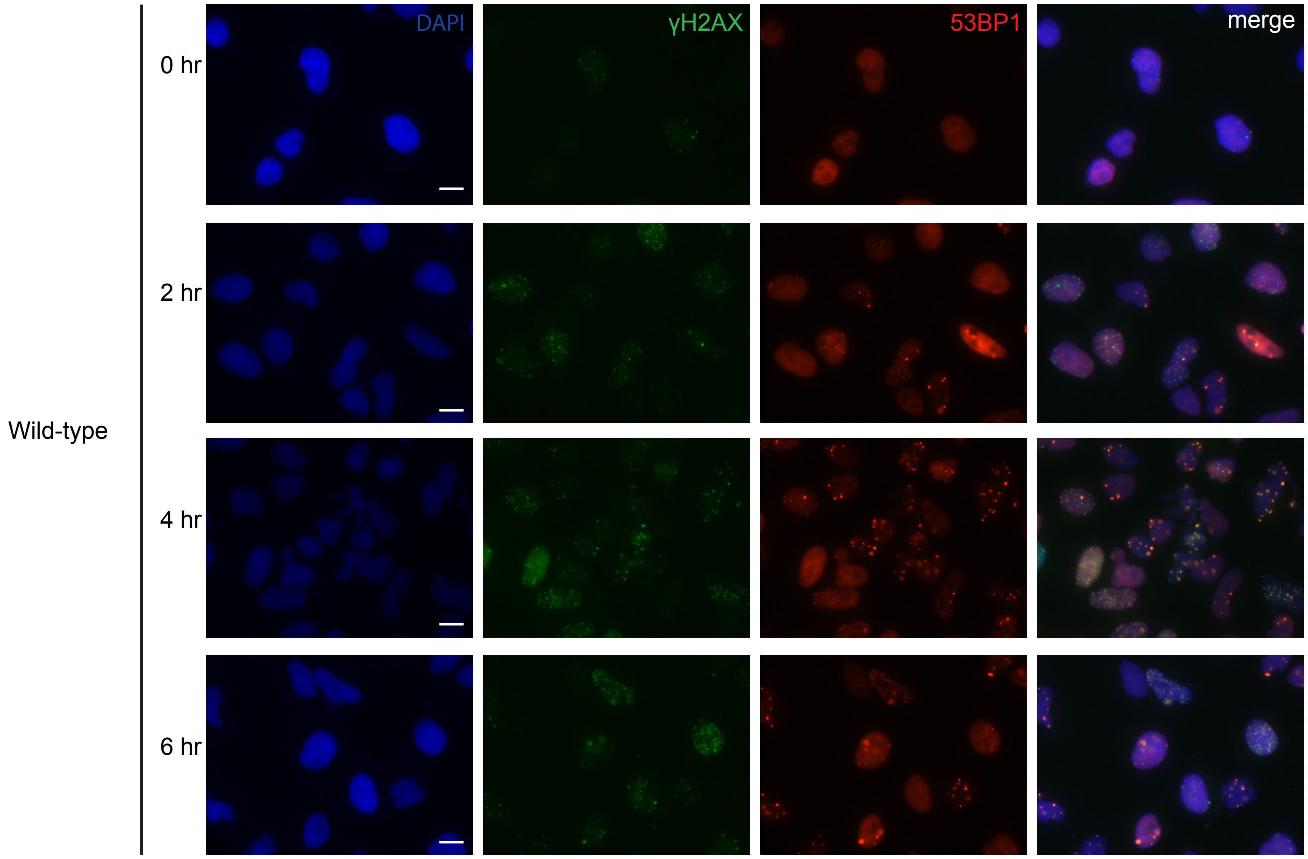

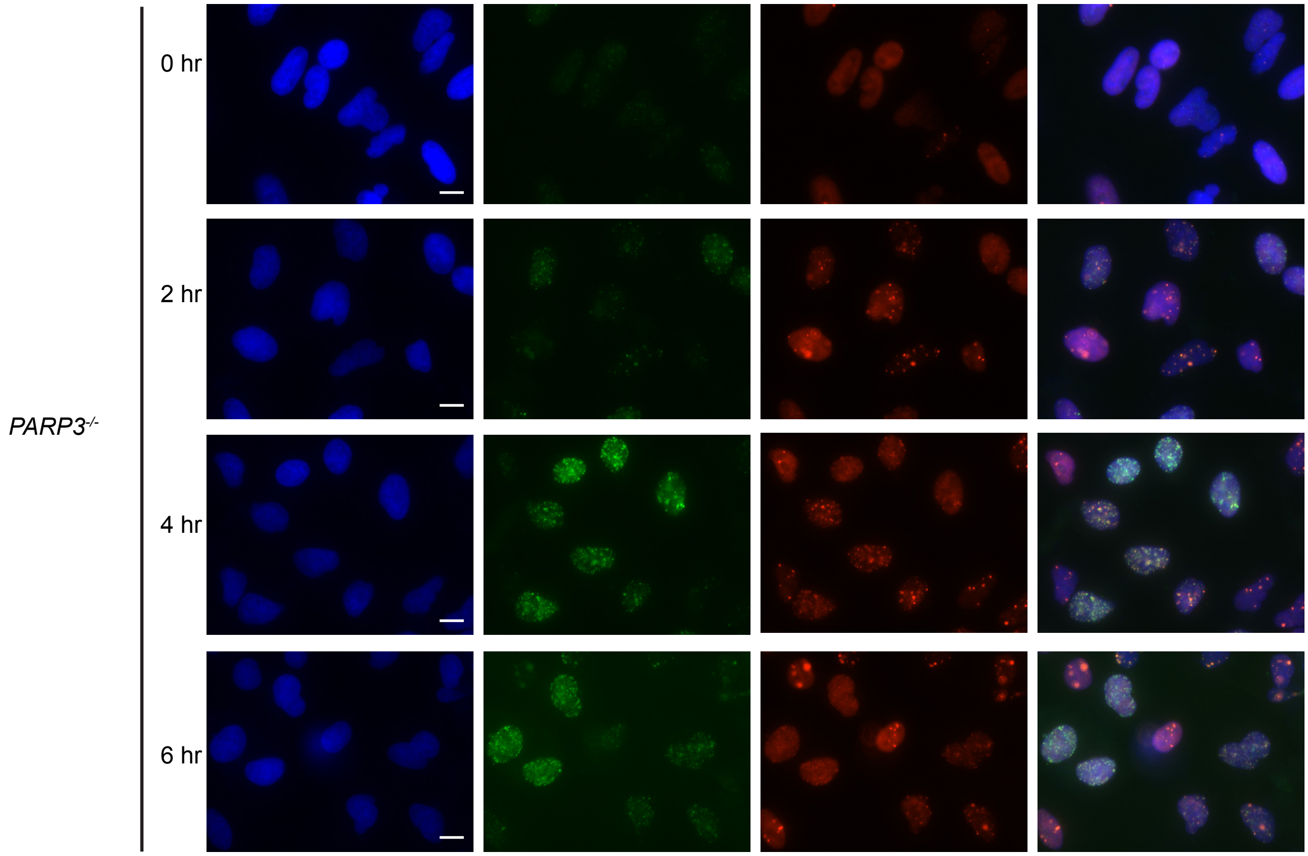

Supplementary Figure 11 | Representative immunofluorescence (IF) images of DAPI, γH2AX, 53BP1, and the merged image at the indicated time points following 5 μM pyridostatin treatment in wild-type and *PARP3*$^{-/-}$ A549 cells. Scale bar, 10 μm.

Supplementary Figure 12

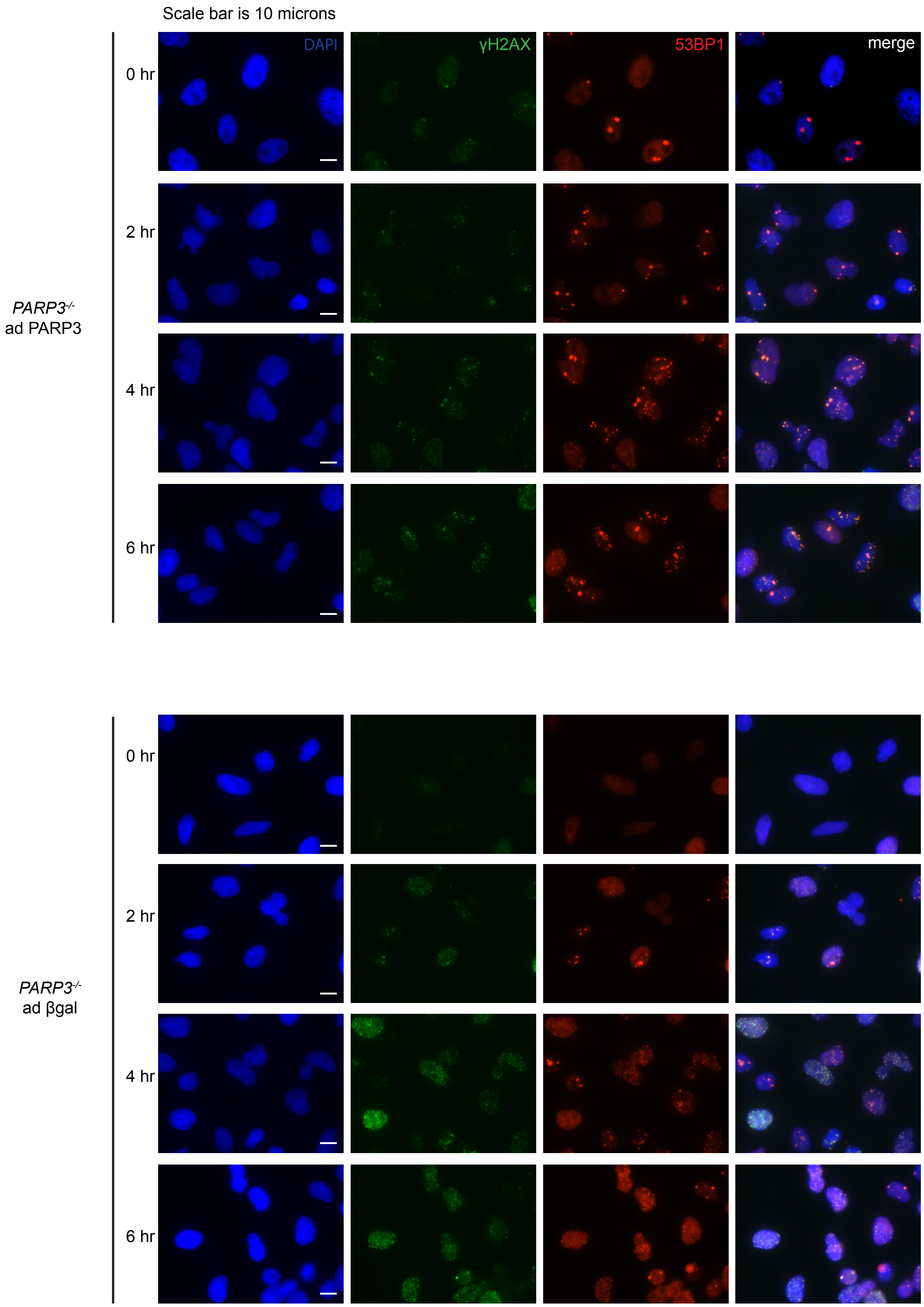

Supplementary Figure 12 | Representative immunofluorescence (IF) images of DAPI, γH2AX, 53BP1, and the merged image at the indicated time points following 5 μM pyridostatin treatment in *PARP3*$^{-/-}$ A549 cells infected with adenovirus expressing PARP3 (ad PARP3) or β-galactosidase (ad β-gal). Scale bar, 10 μm.

Supplementary Figure 13

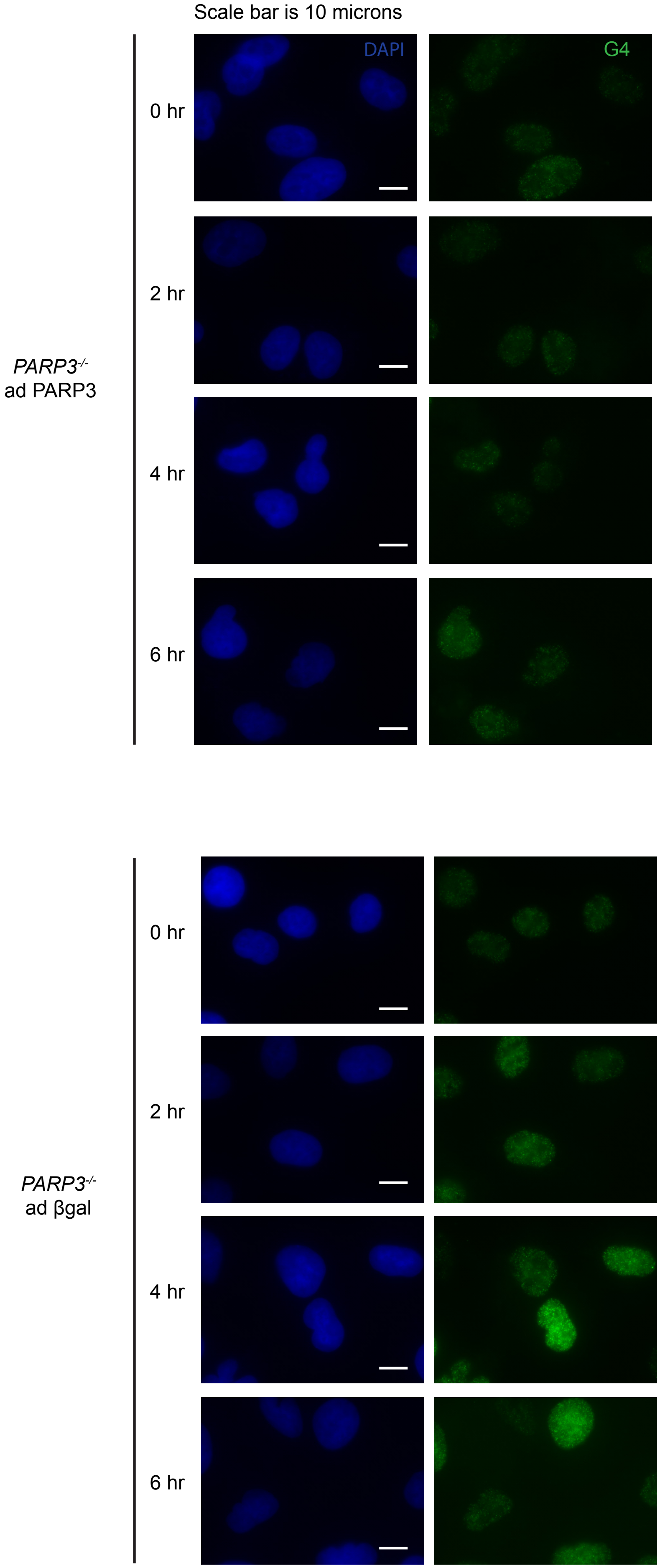

Supplementary Figure 13 | Representative immunofluorescence (IF) images of DAPI and 1H6 antibody (or G4 DNA) staining at the indicated time points following 5 μM pyridostatin treatment in *PARP3*$^{-/-}$ A549 cells infected with adenovirus expressing PARP3 (ad PARP3) or β-galactosidase (ad β-gal). Scale bar, 10 μm.

Supplementary Figure 14

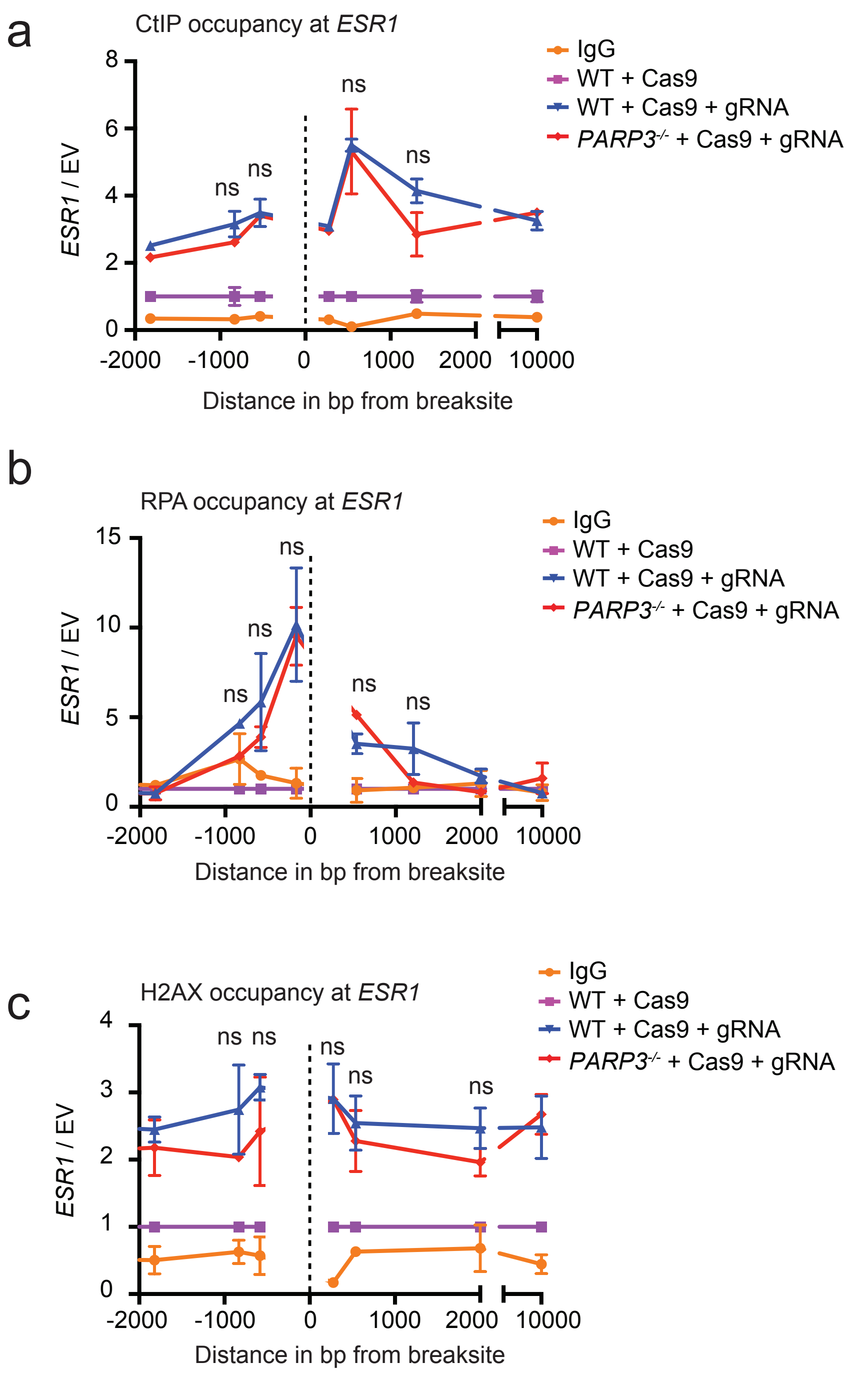

Supplementary Figure 14 | a-c CtIP (a), RPA (b), and γH2AX (c) occupancy at the ESR1 locus measured by ChIP in the indicated genetic backgrounds 18 hours after transient expression of Cas9 alone or with gRNA targeting ESR1. bp, base pairs. Data represents mean ± SE.

Supplementary Figure 15

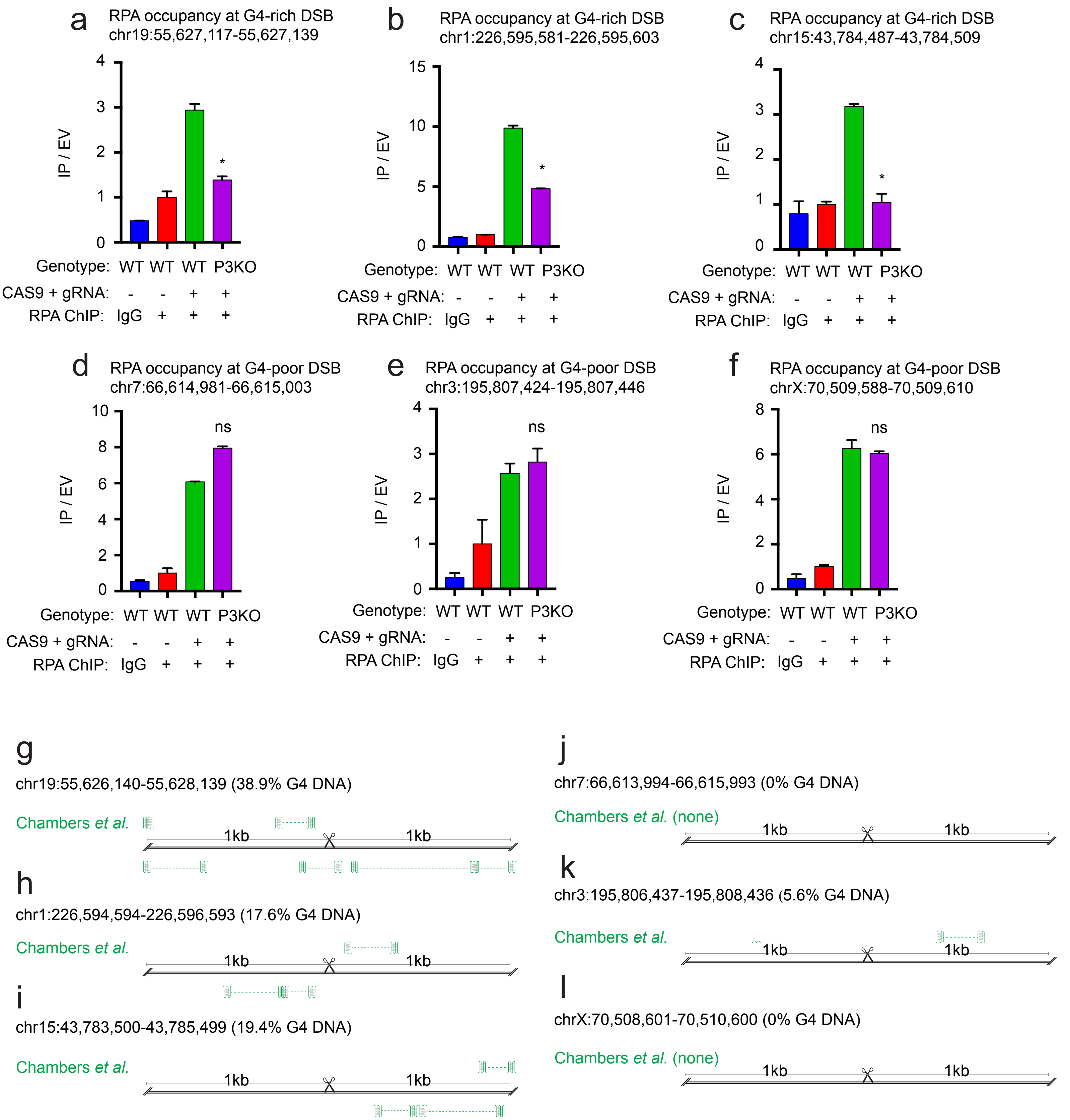

Supplementary Figure 15 | a-f RPA occupancy at three G4-rich loci (**a-c**) and three G4-poor loci (**d-e**) measured by ChIP in the indicated genotypes. See **Supplementary Tables 5 and 7** for gRNA sequences and ChIP primers. WT, wild-type. P3KO, *PARP3*$^{-/-}$. *P* values calculated using unpaired Student's t test. **P* < 0.05, ***P* < 0.01, ****P* < 0.001. Data represents mean ± SE. **g-l** Schematic of sequences experimentally observed to form G4 DNA by Chambers *et al.*, 2015. (**g-i**) G4-rich sequences. (**j-l**) G4-poor sequences.

Supplementary Figure 16

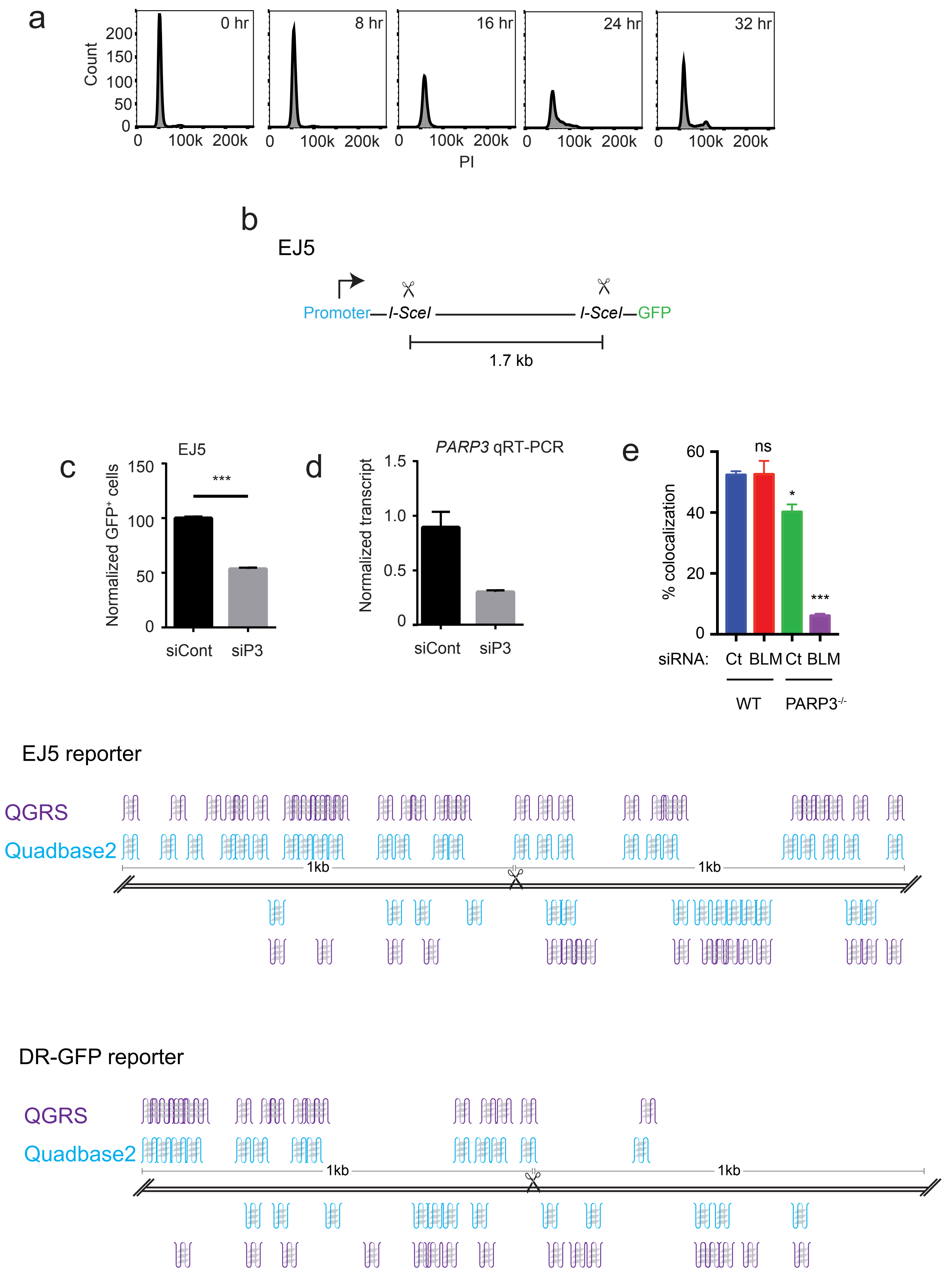

Supplementary Figure 16 | (**a**) Propidium iodide (PI) content of serum-starved A549 cells released into 20% serum for the indicated times. (**b**) Schematic of the EJ5 end joining reporter. Cleavage of both sites by the I-*Sce*I endonuclease and deletion of the 1.7 kb intervening sequence results in expression of the *GFP* gene from the upstream promoter, which can be quantified by flow cytometry. (**c**) Normalized frequency of GFP-positive cells in HeLa cells containing EJ5 transfected with siRNA targeting PARP3 (siP3) or Control (siCont). (**d**) Quantification of PARP3 transcript 24 hours after transfection with siRNA targeting PARP3 (siP3) or Control (siCont). (**e**) Degree of colocalization between γH2AX and 53BP1 at 1 hour following 10 Gy ionizing radiation in wild-type (WT) and $PARP3^{-/-}$ A549 cells treated with siRNA targeting non-targeting control (Ct) or BLM. P values calculated using unpaired Student's t test. $^{*}P < 0.05$, $^{**}P < 0.01$, $^{***}P < 0.001$. Data represents mean ± SE. (**f,g**) Sequences predicted by QGRS (purple) and Quadbase2 (blue) to form G4 DNA in a 2000 base pair window surrounding the I-*Sce*1 sites in the EJ5 reporter (**f**) and the DR-GFP reporter (**g**). Scissors, CRISPR/Cas9 cut site.

Supplementary Figure 17
Whole portions of western blots

Figure 2a: RAD50

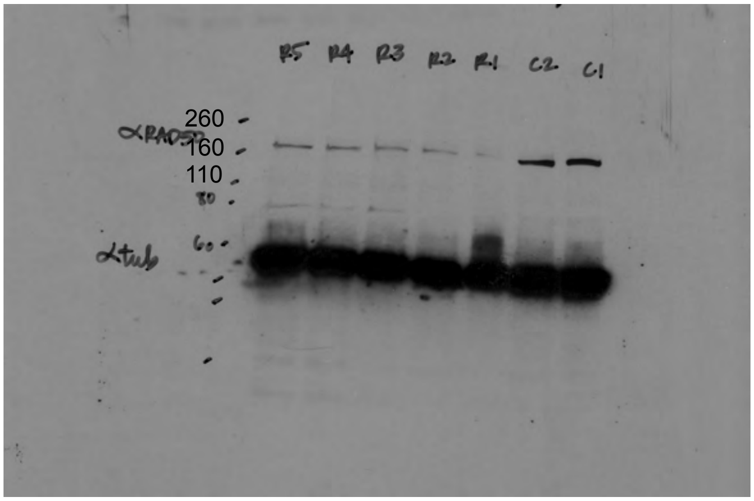

Figure 2b: UBC9

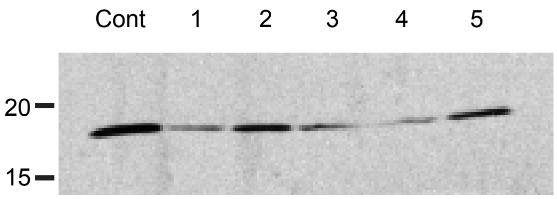

Figure 2c: DDB1

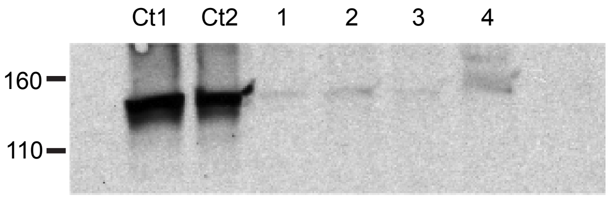

Figure 2d: 53BP1

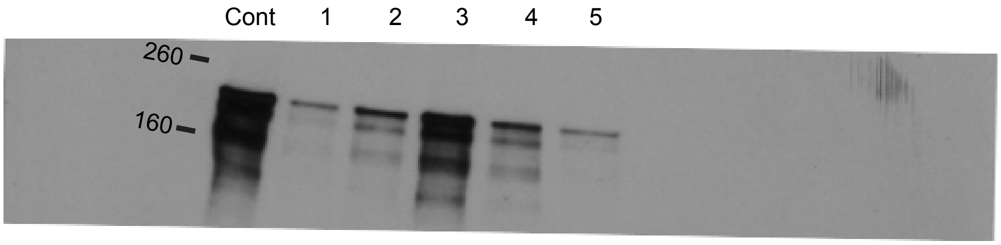

Figure 2e: PARP3

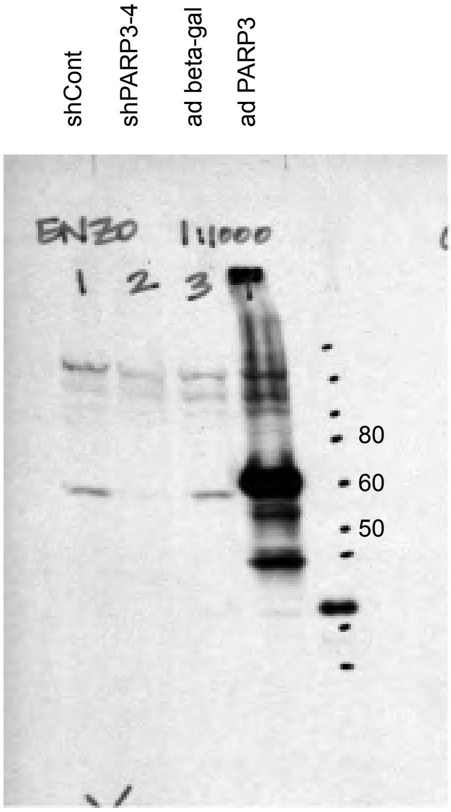

Figure 3g: PARP3

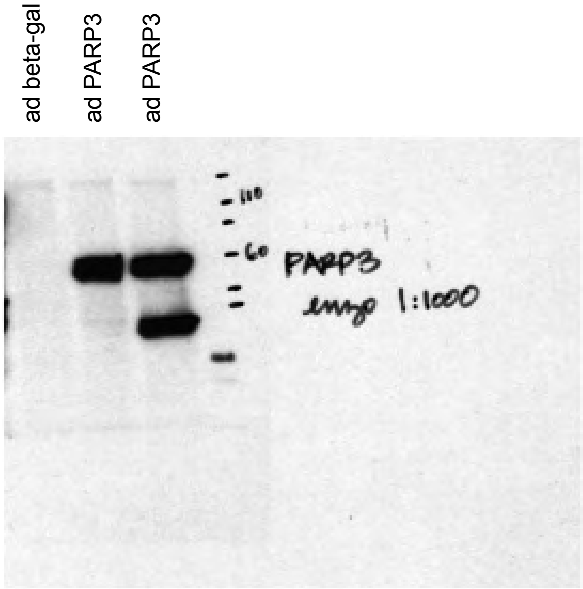

Supplementary Table 1. List of differentially expressed genes with adjusted p-value<0.05 and $\log_2$ fold-change >1 of relative log-transformed values across samples vehicle-treated PARP3$^{-/-}$ vs. vehicle-treated wild-type A549 cells (N=61)

	$\log_2$FoldChange	standard error	adjusted-p-value
CPS1	-2.870670458	0.084374332	1.55E-249
PTPRD	-2.544686444	0.111347332	2.92E-112
CYP4F11	-2.474712512	0.110397899	4.61E-108
H19	-2.245936667	0.098387106	4.64E-112
FGB	-1.857667459	0.097912653	2.56E-77
PLXDC2	-1.637847767	0.121265723	4.28E-39
FILIP1	-1.570134846	0.104652437	2.95E-48
SPINK6	-1.498997009	0.121580852	1.47E-32
SUSD2	-1.488075679	0.064499884	2.30E-114
FGA	-1.487267913	0.096836988	1.48E-50
PDE3A	-1.474106086	0.095523849	4.90E-51
NOTCH3	-1.461923943	0.092941183	5.15E-53
CES1	-1.446777121	0.121199379	1.65E-30
SMOC1	-1.430452701	0.12076914	4.85E-30
AQP3	-1.410391366	0.094759287	1.68E-47
CEACAM5	-1.406694081	0.099720843	1.23E-42
DLC1	-1.395379189	0.110836247	6.01E-34
FGG	-1.345218664	0.122002186	4.72E-26
RTN4RL2	-1.305900875	0.094007735	2.41E-41
PPARGC1A	-1.287255046	0.119340781	6.07E-25
SLC2A3	-1.23338582	0.068373433	7.32E-70
MUC5B	-1.229529761	0.113287899	2.99E-25
CYP1B1	-1.225350524	0.080547675	1.30E-49
GREM2	-1.155715419	0.122179638	3.75E-19
AMOT	-1.14454514	0.122100494	7.82E-19
ABCB1	-1.132309165	0.120543931	6.75E-19
NPR3	-1.11301971	0.101691195	1.12E-25
RSPO3	-1.101745956	0.072803365	4.25E-49
VCAN	-1.09853804	0.09340993	1.30E-29
DEFB1	-1.068596158	0.120317925	6.83E-17
EDIL3	-1.035046494	0.122285832	2.42E-15
GPR37	-1.015952387	0.093405815	2.33E-25
NTRK3	-1.008305292	0.06297273	5.95E-55
SAMD11	-1.001447359	0.089832644	1.27E-26
VNN1	1.004396205	0.118570574	2.30E-15
CRISPLD2	1.013711401	0.088470675	4.02E-28
FAM189A1	1.023783266	0.109012777	6.82E-19
RASGRF2	1.04466433	0.082824807	4.54E-34
DPYSL3	1.044981728	0.039011987	2.37E-154
KLF12	1.050426928	0.114537364	5.09E-18
COL6A1	1.091731508	0.056976953	7.45E-79
IL7R	1.150317631	0.078389851	3.67E-46
CCBE1	1.200117338	0.102055173	1.30E-29
CSF2	1.200555278	0.112947176	3.24E-24
NID1	1.214570864	0.116143378	2.00E-23
PTHLH	1.237361831	0.085655646	9.88E-45
COL6A2	1.248866387	0.07177107	5.16E-65
PLAT	1.385358969	0.102000025	1.59E-39
ST6GAL2	1.392177093	0.096110913	5.72E-45

ANXA10	1.436378036	0.080528066	2.52E-68
MECOM	1.460191651	0.119913814	9.21E-32
KIAA1462	1.493249132	0.088972925	1.97E-60
ABLIM3	1.593892972	0.066964494	9.67E-122
UGT8	1.624078789	0.120813207	9.91E-39
ANPEP	1.640270228	0.088090708	1.76E-74
ADAM19	1.726042393	0.081849871	1.56E-95
SPOCK1	1.788355709	0.114493062	2.80E-52
SPARC	1.909453696	0.098381245	6.60E-81
RRAD	1.963100423	0.080572431	1.55E-127
UPK1B	2.06610896	0.110901529	1.55E-74
PXDN	2.538435519	0.094047121	1.43E-156

Supplementary Table 2. List of differentially expressed genes with adjusted p-value<0.05 and log_2 fold-change >1 of relative log-transformed values across samples pyridostatin-treated PARP3$^{-/-}$ vs. pyridostatin-treated wild-type A549 cells (N=64).

	log_2FoldChange	standard error	adjusted-p-value
CPS1	-3.024545179	0.08432471	1.44E-277
PTPRD	-2.788062107	0.110532868	8.14E-137
CYP4F11	-2.535549093	0.109890818	1.82E-114
H19	-2.224701699	0.101354144	1.29E-103
FGB	-1.825824555	0.098336042	4.18E-74
PLXDC2	-1.80925528	0.120692553	3.39E-48
FILIP1	-1.655926954	0.102289386	3.10E-56
SMOC1	-1.643358083	0.119664092	2.03E-40
CES1	-1.495773953	0.120779744	7.14E-33
SUSD2	-1.463803315	0.063540678	3.66E-114
SPINK6	-1.433765778	0.121486416	7.87E-30
CEACAM5	-1.40299113	0.100242171	5.56E-42
NOTCH3	-1.389977166	0.092531081	2.18E-48
DLC1	-1.364275172	0.109552897	3.07E-33
PDE3A	-1.339880416	0.094570765	4.99E-43
FGA	-1.339639847	0.096073066	1.13E-41
PPARGC1A	-1.33018542	0.118330082	4.46E-27
AQP3	-1.323905051	0.0928449	1.42E-43
SLC2A3	-1.245258048	0.066658408	5.81E-75
FGG	-1.230293221	0.12195329	8.25E-22
EDIL3	-1.220243785	0.12194278	1.85E-21
AMOT	-1.209774032	0.122041097	4.60E-21
CELF2	-1.180805614	0.119305632	5.33E-21
MUC5B	-1.177655126	0.113234981	3.53E-23
NTRK3	-1.174123834	0.062903465	7.38E-75
ABCB1	-1.172261139	0.120832976	3.53E-20
CYP1B1	-1.141253952	0.080424651	3.71E-43
VCAN	-1.138032133	0.092851915	3.39E-32
CPLX2	-1.095622433	0.046032713	8.07E-122
PDK4	-1.082592671	0.060730297	2.62E-68
GREM2	-1.061144297	0.122128773	3.40E-16
CEMIP	-1.048034657	0.107805192	2.92E-20
HYAL1	-1.034515953	0.113306443	7.09E-18
PCSK9	-1.012504769	0.098524886	1.23E-22
RNF43	-1.011551508	0.066702352	2.63E-49
NT5E	1.043102796	0.051710891	1.83E-87
RASSF10	1.045775794	0.080674106	5.08E-36
CORO2B	1.052529898	0.118099458	4.91E-17
CDH6	1.066224679	0.096359893	3.06E-26
IL7R	1.086468477	0.07930488	3.14E-40
FEZ1	1.111122026	0.119276332	1.32E-18
PLAT	1.120048803	0.100986893	2.31E-26
CSF2	1.13605551	0.112863866	1.03E-21
COL6A1	1.154337935	0.056394006	4.63E-90
VNN1	1.155341932	0.117633227	1.12E-20
KLF12	1.186985145	0.113853844	2.79E-23
MUC3A	1.223058975	0.088273223	3.81E-41
NID1	1.265110777	0.116088394	1.90E-25
PTHLH	1.269924423	0.086344564	2.25E-46
COL6A2	1.344802768	0.071557273	7.47E-76
UGT8	1.352346714	0.120777965	7.28E-27
CCBE1	1.357339211	0.10301212	3.17E-37
ANXA10	1.375878143	0.080239823	3.77E-63
KIAA1462	1.379425032	0.084783851	8.55E-57
ABLIM3	1.472649362	0.065762942	7.54E-108
ST6GAL2	1.480469769	0.094982621	4.15E-52

MECOM	1.59385631	0.118675409	1.17E-38
ANPEP	1.617378492	0.08731495	9.00E-74
ADAM19	1.688663849	0.08055274	1.75E-94
SPOCK1	1.756107796	0.112641701	4.06E-52
SPARC	1.862520814	0.098197688	2.96E-77
UPK1B	2.00321143	0.110306753	6.86E-71
RRAD	2.165775082	0.079540503	1.47E-159
PXDN	2.534107973	0.092002424	3.88E-163

Supplementary Table 3. CRISPR/Cas9 RNA guide sequences.

Target	5'-3' Sequence (PAM)
Human PARP3 RNA guide 1	GGACTGAGAGTGGGTCGTTG(GGG)
Human PARP3 RNA guide 2	TGTACCCAGGGCTTCGGCTT(TGG)
Human LigIV RNA guide 1	GTGCTATGTGGTAGTCTGAC(AGG)
Human LigIV RNA guide 2	AATGCACACATAGTATCGCA(TGG)
Human 53BP1 RNA guide 1	CAGGTTCTAGAGGATGATTC(TGG)
Human 53BP1 RNA guide 1	CAGGATTTTCTTTGTGCGTC(TGG)
CD4 RNA guide for ChIP	CAGAGGTGTCTTACCCTAG(GGG)
ESR1 RNA guide for ChIP	AGAACGCTCATTGTGTCGTT(TGG)
G4 rich chr19:55,627,127-55,627,149	GGGGCCACTAGGGACAGGAT(TGG)
G4 rich chr1:226,595,581-226,595,603	CGAGTCGAGTACGCCAAGAG(CGG)
G4 rich chr15:43784487-43784509	CAGGATTTTCTTTGTGCGTC(TGG)
G4 poor chr7:66,614,981-66,615,003	GTAATTTGCTTTCATAAGCC(AGG)
G4 poor chr3:195,807,424-195,807,446	ACTAGCATTGTGATCGATTC(AGG)
G4 poor chrX:70,509,588-70,509,610	ACTACGGCACCACTATGCCT(GGG)

Supplementary Table 4. qRT-PCR and qPCR primers.

Primer	5'-3' sequence
hPARP3 FOR	CCTCTGTCCACCTTCACCAC
hPARP3 REV	CATCCAGCTGCTCCAAGACA
hPARP3 FOR in CRISPR deletion	CATGGCTCCAAAGCCGAAGC
hPARP3 REV in CRISPR deletion	CCTTGCTCTGGCGCTTACC
hBeta-Actin FOR	GATGCAGAAGGAGATCACTGC
hBeta-Actin REV	CTTCCAGCAGATGTGGATCA
293T cutting control FOR	AGGGAGCTGCAGGCTGG
293T cutting controls REV	TGCTTGCTTTGGTGCTTTGG
HeLa cutting control FOR	CGGTTAATGTGGCTCTGGTT
HeLa cutting control REV	ACAGGAGGTGGGGGTTAGAC

Supplementary Table 5. ChIP primers.

CD4 ChIP primers

Primer	5'-3' sequence
-100000 FOR	TTGGGGACTGAGAGGTGACT
-100000 REV	AAGCTGCCCTGCTTGGTTTA
-5000 FOR	TGTTGTCCGAGCAAGGGATG
-5000 REV	CCCCCTTCACCCAACTTCTC
-2000 FOR	CTTTCCGCCCTCAGACCTTT
-2000 REV	ATGTCCACCACAAGAGCAGG
-1005 FOR	CTTTGATGAAGGTGGGGAATCA
-1005 REV	GCCATCCTCTTCTTCGCCTC
-703 FOR	GCAGTAGAGAGGGTGAACGC
-703 REV	CAACACACACCACGCACTTT
-523 FOR	TGAGAAAGTGCGTGGTGTGT
-523 REV	GAGACCTGGAGCAGCTTGTT
-296 FOR	TTTGTCCATGTGTCCCTCCC
-296 REV	TCTAGCTCCTTCCGGTCTGT
197 FOR	GGAGCTGGGGGAGTCAAAAC
197 REV	CTGACTGGGTCCTTGGTGAG
287 FOR	TTGGACCCCAGAGAAGGGAT
287 REV	TTAGCATGCAGATGGGAGGC
517 FOR	GAAACCTCCTTCCTGACCTCTT
517 REV	TGGGCGACAGAGTGAGATTTTG
2000 FOR	GGCAGATGGAGTAACTGGAGG
2000 REV	TGAACCATGGGAGGTGGACT
5000 FOR	GGGTCCACGTGGCAAATCTA
5000 REV	ATGCATGGAGAAGGTGGCAA
100000 FOR	GTCCCCGCCAATCTCTTCTC
100000 REV	CTCTCTTCTGGAACCCGAGC

ESR1 ChIP primers

-100000 FOR	TCTTCGCAGTTTGCAGTTTG
-100000 REV	CCCTTAGACAAGCGCAAGAA
-1820 FOR	CCCGAGTAGCTGGGATTACA
-1820 REV	CCTGAGGTCAGGAGTTCGAG
-831 FOR	AGAAAGGGCCAGAAACATGA
-831 REV	CCAGATTTAGACCCACCTGCT
-581 FOR	TACGAGGGCCGTGCTAAT
-581 REV	GGCAGGTGGATATGTTGAAA
-168 FOR	CGACCAGGCACAGATGTAAG
-168 REV	CTGACCTCCTGATGGACTCA
271 FOR	TTGCTGTGTCCTCACATGGT
271 REV	GCCTTTGGTGGGTGATTAGA
541 FOR	GACTGTTTGGGTGGCATTCT
541 REV	CTGGGGACAATGGTTGAAAT
1308 FOR	TTCCCTACCTCATGCAAACC
1308 REV	GTCTGCCAGCTGCAATATCA
2000 FOR	AGGTTGCAGTTGGGTAGTGG
2000 REV	CTCACGATGGCAGGGTTATT
100000 FOR	TAGGGCAGGCATGGTTATTC
100000 REV	CATTCTCTCTCGCGCTCTCT

Other ChIP primers

TEL FOR	GGTTTTTGAGGGTGAGGGTGAGGGTGAGGGTGAGGGT
TEL REV	TCCCGACTATCCCTATCCCTATCCCTATCCCTATCCCTA
ESR1 FOR	GAAACAGCCCCAAATCTCAA
ESR1 REV	TTGTAGCCAGCAAGCAAATG
MYC FOR	CCTTAGCATTCGCTTTCCAG
MYC REV	AGTCTGGGTGTGGGCATAAG
chr8 FOR	TCCTTTGATGAATGGCTGAA
chr8 REV	GAACTTTGCTCCTGCTTCCT
chr22 FOR	CACCGGTAATGAGACCAATG
chr22 REV	GGGAGATTGGGTCTTTCTCA
G4 rich chr19 FOR	GTCCAGGCAAAGAAAGCAAG
G4 rich chr19 REV	TGGGTTCCCTTTTCCTTCTC
G4 rich chr1 FOR	CTGATGTTGCAGGAAAAGCCC
G4 rich chr1 REV	GGCTCCTCGTTTTCACAAAGC
G4 rich chr15 FOR	AAACGGGACCACTTCAGGAC
G4 rich chr15 REV	AGGGAGGGATAGTCAGGCTT
G4 poor chr7 FOR	AGAGGAAAGCTAGAGACTGCC
G4 poor chr7 REV	GCACCTCCACTAAGAGATGGG
G4 poor chr3 FOR	ACAGAATCCAGCCCCTGTTT
G4 poor chr3 REV	GGACGGGAAAAGATTCCTGGT
G4 poor chrX FOR	CACGGTATGTACTGTGGGCT
G4 poor chrX REV	TCGCTTAAGGAACTTCCCAGT

Supplementary Methods

Antibodies

Primary antibodies used in this study were as follows: mouse anti-KU70 (E-5, sc-17789) [1]; rabbit anti-UBC9 (D26F2, 4786, Cell Signaling) [2]; rabbit anti-RAD50 (3427, Cell Signaling) [3]; rabbit anti-53BP1 (A300-272A, Bethyl Labs) [4]; rabbit anti-DDB1 (A300-462A, Bethyl Labs) [5]; rabbit anti-PARP3 (ALX-210-971-R100, Enzo Life Sciences) [6]; rabbit anti-PARP3 (NBP1-31415, Novus Biologicals) [7]; rabbit anti-RPA32 (A330-244A, Bethyl Labs) [8]; rabbit anti-DNA Ligase IV (H-300, Santa Cruz) [9]; rabbit anti-HA (Y-11, Santa Cruz) [10]; mouse anti-DNA G-quadruplex (G4), (1H6, EMD Millipore) [11]; rabbit anti-PARP1 (9542, Cell Signaling) [12]; rabbit anti-BLM (A300-110, Bethyl Labs) [13]; goat anti-PARP2 (A-18, sc-30622, Santa Cruz) [14], rabbit anti-beta-actin (4967, Cell Signaling) [15]. All antibodies were used at 1:1000 dilutions with the exception of the PARP2 antibody which was used at 1:250. All western blots verifying knockdown were repeated for each experimental replicate.

RNA isolation

Total RNA was isolated from wild-type and *PARP3*$^{-/-}$ A549 cells that were treated for 24 hours with either vehicle (DMSO) or 1.0 μM pyridostatin using the Qiagen RNeasy Mini Kit according to the manufacturer's specifications. All RNA-seq experiments were performed in biological triplicate. RNA yield and purity was obtained on a NanoDrop 2000 (Thermo Fisher Scientific).

RNA sequencing

For RNA-seq experiments, RNA was subjected to Illumina-based RNA-seq using a TruSeq Stranded mRNA Library Prep Kit (Illumina) as per the manufacturer's recommendations. RNA-seq libraries were sequenced with an Illumina NextSeq 500 using single end 75 base pair sequencing.

Analysis of RNA-seq data

The R package DESeq2[81] was used to determine differential expression between samples. Rows of data summing to 0 or 1 were removed prior to testing. Wald P-values were adjusted for multiple comparisons by the method of Benjamini and Hochberg to estimate the false discovery rate (FDR) and were considered significant if <0.05. The heatmaps were generated using the pheatmap package using the relative values that were transformed using the regularized logarithm (rlog) function in R. Only genes with a $|\log_2$ fold change$|>1$ and $p_{adjusted} <0.05$ are included in the heatmaps.

Supplementary References

1. Xu, X. et al. The CUL7 E3 ubiquitin ligase targets insulin receptor substrate 1 for ubiquitin-dependent degradation. *Mol Cell* **30**, 403-14 (2008).

2. Hsieh, Y.L. et al. Ubc9 acetylation modulates distinct SUMO target modification and hypoxia response. *EMBO J* **32**, 791-804 (2013).
3. Martin, R.M. et al. Post-transcriptional regulation of MRE11 expression in muscle-invasive bladder tumours. *Oncotarget* **5**, 993-1003 (2014).
4. Wang, J.Y., Chen, S.Y., Sun, C.N., Chien, T. & Chern, Y. A central role of TRAX in the ATM-mediated DNA repair. *Oncogene* (2015).
5. Li, Y., Jaramillo-Lambert, A., Hao, J., Yang, Y. & Zhu, W. The stability of histone acetyltransferase general control non-derepressible (Gcn) 5 is regulated by Cullin4-RING E3 ubiquitin ligase. *J Biol Chem* **286**, 41344-52 (2011).
6. Rulten, S.L. et al. PARP-3 and APLF function together to accelerate nonhomologous end-joining. *Mol Cell* **41**, 33-45 (2011).
7. Galanty, Y. et al. Mammalian SUMO E3-ligases PIAS1 and PIAS4 promote responses to DNA double-strand breaks. *Nature* **462**, 935-9 (2009).
8. Chen, X. et al. 14-3-3 proteins restrain the Exo1 nuclease to prevent overresection. *J Biol Chem* **290**, 12300-12 (2015).
9. Tichy, E.D. et al. Mouse embryonic stem cells, but not somatic cells, predominantly use homologous recombination to repair double-strand DNA breaks. *Stem Cells Dev* **19**, 1699-711 (2010).
10. Ginter, T. et al. Arginine residues within the DNA binding domain of STAT3 promote intracellular shuttling and phosphorylation of STAT3. *Cell Signal* **26**, 1698-706 (2014).
11. Henderson, A. et al. Detection of G-quadruplex DNA in mammalian cells. *Nucleic Acids Res* **42**, 860-9 (2014).
12. Fan, L. et al. Regulation of c-Myc expression by the histone demethylase JMJD1A is essential for prostate cancer cell growth and survival. *Oncogene* **35**, 2441-52 (2016).
13. Tomimatsu, N. et al. Phosphorylation of EXO1 by CDKs 1 and 2 regulates DNA end resection and repair pathway choice. *Nat Commun* **5**, 3561 (2014).
14. Menissier de Murcia, J. et al. Functional interaction between PARP-1 and PARP-2 in chromosome stability and embryonic development in mouse. *EMBO J* **22**, 2255-63 (2003).
15. Wei, F., Scholer, H.R. & Atchison, M.L. Sumoylation of Oct4 enhances its stability, DNA binding, and transactivation. *J Biol Chem* **282**, 21551-60 (2007).
